# Mechanism of down regulation of Na-H exchanger-2 in experimental colitis

**DOI:** 10.1371/journal.pone.0176767

**Published:** 2017-05-11

**Authors:** Amal Ali Soleiman, Farook Thameem, Islam Khan

**Affiliations:** Department of Biochemistry, Faculty of Medicine, Kuwait University, Jabriya, Kuwait; Cairo University Faculty of Pharmacy, EGYPT

## Abstract

**Background:**

The Na-H exchanger [NHE] performs an electroneutral uptake of NaCl and water from the lumen of the gastrointestinal tract. There are several distinct NHE isoforms, some of which show an altered expression in the inflammatory bowel diseases (IBD). In this study, we examined a role of NHE-2 in experimental colitis.

**Methods:**

Colitis was induced in male Sprague-Dawley rats by intra-rectal administration of trinitrobenzenesulphonic acid (TNBS). On day 6 post-TNBS, the animals were sacrificed, colonic and ileal segments were taken out, cleaned with phosphate buffered saline and used in this study.

**Results:**

There was a significant decrease in the level of NHE-2 protein as measured by ECL western blot analysis and confocal immunofluorescence microscopy. The levels of NHE-2 mRNA and heteronuclear RNA measured by an end-point RT-PCR and a real time PCR were also decreased significantly in the inflamed colon. However, there was no change in the level of NHE-2 protein in response to *in vitro* TNF-α treatment of uninflamed rat colonic segment. These changes were selective and localized to the colon as actin, an internal control, remained unchanged. Confocal immunofluorescence microscopy revealed co-localization of NHE-2 and NHE-3 in the brush borders of colonic epithelial cells. Inflamed colon showed a significant increase in myeloperoxidase activity and colon hypertrophy. In addition, there was a significant decrease in body weight and goblet cells’ mucin staining in the TNBS treated colon. These changes were not conspicuous in the non-inflamed ileum.

**Conclusions:**

These findings demonstrate suppression of NHE-2 expression on the brush borders in the colonic epithelial cells which is regulated transcriptionally. However a role of TNF-α in the regulation of NHE-2 is discounted in the present model of colitis. This decrease in the NHE-2 expression will lead to a loss of electrolyte and water uptake thus contributing to the symptoms associated with IBD.

## Introduction

Na^+^/H^+^ exchanger [NHE] is a transmembrane protein that catalyzes an electro neutral exchange of extracellular Na^+^ for intracellular H^+^ [1–4]. It plays an important role in the regulation of intracellular pH, cell volume and cell size. It contributes to a neutral uptake of NaCl and water in the intestine and renal system [1–7]. Among ten different NHE isoforms, the isoforms NHE-2, NHE-3, and NHE-8 are localized to the apical membrane of epithelial cells and play a major role in the absorption of NaCl from the GI lumen [8–16]. The NHE-3 isoform is predominantly expressed in the ileum while NHE-2 is prevalent in the colonic mucosa and crypt cells [15,16]. Although NHE-2 and NHE-3 contribute ∼50% exchange activity to ileal Na^+^/H^+^, apical uptake of Na^+^ in the colon has been attributed to NHE-2 activity which is abundant in the colonocytes and in crypt cells [11,14,16].

Rat NHE-2 polypeptide consisting of 813 aa is broadly organized into two major domains; the N-terminus region [1–500 aa] and a regulatory C-terminus region [501–813 aa] having potential sites for O-linked glycosylation [15]. The NHE-2 isoform has been shown to yield two protein bands; the 85 kD an O-glycosylated, and a 75 kD as an unglycosylated NHE-2 protein [13–16]. Unlike NHE-3, the isoform NHE-2 does not recycle between the plasma membrane and endosomes under different physiological conditions [16].

Various cis-acting elements have been identified in the NHE-2 gene promoter which regulate its expression under different conditions [17]. For example, a nuclear factor *kappa*-B [NF-kB] binding site makes it respond to proinflammatory cytokines, infectious agents, and oxidative stress. Therefore, high levels of pro-inflammatory cytokines, such as interferon-γ [IFN-γ], tumor necrosis factor-α [TNF-α] in IBD might regulate NHE-2 expression leading to alteration in the secretion and absorption of electrolytes in the inflamed colon [17–19]. It is interesting to note that TNF-α decreases the expression and activity of NHE-2 in the intestinal epithelial cell line, C2BBe1 [17]. This is consistent with previous investigations which have shown a strong reduction in Na^+^ absorption in IBD patients [20–21]. Therefore it is possible to speculate that altered expression and activity of NHE-2 could cause IBD, and that this abnormality is regulated by TNF-α. Since both secretion and absorption of electrolytes are altered in IBD, our hypothesis is that NHE-2 expression should alter in response to inflammation in rat colon.

In this study, we used an animal model of IBD induced in male Sprague-Dawley rats by intra rectal administration of TNBS. The method of colitis induction in rat is well established in this laboratory, and the present model has shown characteristics that mimic features of the human IBD. In this study tissues from day 6 post-TNBS colitis were used.

## Materials and methods

### Induction of colitis

Male Sprague-Dawley rats weighing 200–250 gm obtained from the Faculty of Medicine Animal Facility were used in this study. The animals were housed in a 12-hourly day and night cycle and provided with free access to normal food and water. The animals were treated in accordance with the animal care guidelines of the Faculty of Medicine, Kuwait University. The animals were divided into two groups; the colitis group in which each animal received trinitrobenzenesulphonic acid, TNBS [Fluka, Co] solution containing 30 mg TNBS dissolved in 250 μl of 50% ethanol intra-rectally about 8 cm from the anal margin, and the control group in which each animal received phosphate buffered saline [PBS] in a similar fashion [22–25]. The body weights of the animals were recorded prior to sacrifice on the day 6 post-TNBS administration. The animals were sacrificed by cervical dislocation. Colonic and ileal segments were taken out, and cleaned with PBS prior to use. In this study we used distal segments of the colon and ileum. The protocol was approved by the Committee on the Ethics of Animal Experiments of Kuwait University Faculty of Medicine.

### Characterization of colitis

Colitis was characterized by measuring myeloperoxidase activity [MPO] following standard methods [22–25]. Tissue segments were minced using a polytron [Brinkman Instruments Co., Westbury, NY], the MPO activity was measured in clarified tissue lysates, and expressed as units per mg tissue [22–25]. MPO activity unit was defined as nmoles of H_2_O_2_ converted to water per min per mg tissue.

### Body weight and colon hypertrophy

Body weight [BW] of each animal was recorded before induction of colitis on day 0 and on day 6 post-TNBS just before sacrifice. Changes in the BW on day 6 were calculated with respect to their initial BW at day 0. Colon length and weights without feces were also measured on day 0 and day 6 post-TNBS, and colon hypertrophy was expressed as weight [mg] per cm length.

### Histochemistry and goblet cell staining

Standard methods were used to fix tissues in paraformaldehyde and for embedding in paraffin blocks [24]. Colon sections of 7 μm thickness were cut and stained with hematoxylin and eosin dye solutions following standard deparaffinization, dehydration and rehydration steps [24]. For goblet cell staining, an alcian blue dye solution, pH 2.5 was used following standard method [26]. After adding mounting solution, tissue sections were photographed using a microscope attached camera.

### Expression of NHE-2 protein

Tissue segments were chopped finely with scissors using 10 ml [per gm tissue] of ice cold MOPS-sucrose (3 [N-Morpholino]propanesulfonic acid) buffer, pH 7.4 [22–25]. The tissues were homogenized using a polytron and then centrifuged at 1620xg for 10 min in a bench top centrifuge [Beckman]. The supernatants were collected and centrifuged at 10,000xg for 10 min in a high speed centrifuge machine [Beckman, JA-20]. All steps were performed at 4°C in this procedure. Supernatants were collected and protein concentrations were estimated using a dye binding kit following the instructions provided by the supplier [BioRad, Hercules, CA]. A standard curve was prepared simultaneously using bovine serum albumin [Sigma] to calculate protein concentrations in the tissue lysates.

The samples containing 2–3 mg/ml protein were prepared using a 4 x sample buffer, heated in a boiling water bath for 5 min and then transferred directly onto an ice bath before loading onto an 8% polyacrylamide gel. The buffer and gel solutions were prepared following methods as used earlier in this laboratory [22–25]. Lysate proteins were separated along with a prestained protein size marker [BioRad] electrophoretically, and then electroblotted onto a PVDF membrane [Amersham] overnight using 20 mV [22, 24–27]. PVDF membranes were washed for 5 min twice with PBS, and blocked with a 5% fat free milk solution prepared in PBS for 30 min. The membranes were incubated with anti NHE-2 rabbit polyclonal antibodies [Origene] using a dilution of 1:200 overnight at 4°C. According to the data sheet provided by the supplier the anti-NHE-2 primary antibodies are highly selective as their reaction was abolished upon pre-incubation of these antibodies with the immunogenic peptide used to induce these antibodies [Origene]. The membranes were washed again with PBS for 15 min before incubating with a 2° antibody-HRP conjugate [Jackson ImmunoResearch, USA] for 2 hr. After washing thoroughly with PBS, the membranes were incubated with enhanced chemiluminescence reagents 1 and 2 [Amersham Biosciences] and exposed to X-ray film [X-OMAT film, Kodak] to develop specific bands. The band density was estimated using a densitometer [SynGene, Chemi Genius Bio Imaging System]. All steps were performed at room temperature with gentle shaking unless stated otherwise.

### NHE-2 localization and measurement by confocal microscopy

Levels of NHE-2 protein were estimated by measuring the fluorescence units obtained by confocal immunofluorescence microscopy. Paraformaldehyde fixed colonic tissues were embedded in paraffin to prepare paraffin blocks. Tissue sections [7 μm thickness] were placed onto glass slides, deparaffinized, dehydrated and rehydrated using graded ethanol solutions [24]. Sections were subjected to microwave treatment to expose antigenic epitopes, blocked using a 1% BSA solution, and then incubated with the anti-NHE-2 and anti-NHE-3 polyclonal antibodies [1:25 dilution] overnight. Then the sections were washed and incubated with suitable 2°Ab-rhodamine [NHE-2] and 2° Ab-FITC [NHE-3] conjugates together and separately. After staining with DAPI, the sections were mounted with a mounting solution, and visualized under a confocal microscope [Zeiss] using light of appropriate wave lengths. For the measurement of expression levels, 3–4 areas were selected from each field and fluorescence units were obtained for the NHE-2 labeling. These experiments were performed with negative controls in which tissue sections were incubated with 2°Ab conjugates only without the 1° Abs.

### Expression of NHE-2 mRNA

To understand the underlying mechanism of NHE-2 protein expression we quantitated the levels of NHE-2 transcript. Two approaches, an end-point cycle RT-PCR and a SYBR-green real time RT-PCR were used to estimate the level of NHE-2 mRNA. For this purpose upstream [PR1] and downstream [PR2] primers were designed using a published NHE-2 cDNA sequence [Table 1]. The mRNA levels were estimated relative to a competitive control [CC] which was constructed using the primers PR1 and PR3. The bridge-primer [PR3] was selected from 2481–2493 bp positions of the published cDNA sequence [Table 1]. The bridge primer consisted of PR2 sequence placed at 5’ end [underlined], and the remaining sequence which was complementary to the NHE-2 cDNA sequence [Table 1]. The sizes of the NHE-2 target and the CC were expected respectively to be 300 bp and 175 bp [Table 1].

**Table 1:** Shown are primer structure (5’-3’), references and expected PCR fragment size in base pair (bp).

Primers	PCR Band	References
PR1	300 bp	NM_001113335.1
TTCTCAAAGAAAGCCTCACCA		
[2341–2361 bp; Tm 60°C]		
PR2		
TTTGGCTTCATATCATGGCTTT		
[2619–2640 bp; Tm 60°C]		
PR3	175 bp	
TTTGGCTTCATATCATGGCTTTGGAGAGCAGGGGC		
[2481–2493 bp; Tm 46°C]		
PR4 (Hnup)	325 bp	NM_012653
TGCATATAGCAGTTAAAGTAAG		
[47172974–47172995 bp; Tm 58°C]		
PR5 (Hndown)		
TGGTGAGGCTTTCTTTGAGAA		
[47173278–47173298 bp; Tm 58°C]		

PR1 = Upstream primer, PR2 = Downstream primer used for mRNA amplification, and PR3 = Bridge primer used to prepare a competitive control. The underlined sequence in PR3 is PR2 sequence. Hnup/Down = Heteronuclear RNA upstream (PR4) /downstream (PR5) primers.

### Preparation of competitive control

An aliquot of total RNA (1μg) was reverse transcribed using 1 pmol of PR3 primer using a single step RT-PCR kit [Amersham], and subsequently amplified for 28 cycles using the primers PR1 and PR2 [100 pmoles each]. The following cycle settings were used: denaturation [94°C x 30 sec]; annealing [55°C x 30 sec]; extension [74°C x 30 sec]. The PCR product [CC, 175 bp] was separated by the agarose gel electrophoresis, and purified. An optimal concentration of the purified CC measured separately was used to spike each sample for its co-amplification with the NHE-2 transcript. This process was called as competitive RT-PCR method because the same set of primers were used to amplify both the target and the control. In this method variations dependent on the structure and composition of the primers, the target and the control are minimized, and hence this quantitation is more accurate than the normal RT-PCR in which a house-keeping gene is amplified using two different sets of primers.

### Total cellular RNA extraction

Total RNA from rat colon was extracted using a Trizol RNA extraction kit following instructions from the supplier [GIBCO]. Concentration and purity of total RNA were estimated from optical densities obtained at 260 and 280 nm using a spectrophotometer [Pharmacia].

### Agarose gel analysis of total RNA

The quality of total RNA was assessed using a formaldehyde agarose gel electrophoresis and a nano-drop method [RCF, HSC, Kuwait University, Kuwait]. An aliquot of total RNA [5–10 μg] was mixed with an equal volume of 2 x RNA buffer [Ambion] and heated at 80°C for 5 min. The samples were then separated electrophoretically on a 1.4% agarose gel containing 1% formaldehyde, or on 8% polyacrylamide gel as described earlier [22, 24–25]. The gels were stained with ethidium bromide, visualized under UV light and photographed.

### End-Point RT-PCR quantitation of NHE-2 mRNA

Different concentrations of CC were used to obtain an optimal concentration for quantitation. A known optimized concentration of CC was then used to spike both the control and colitis samples, and co-amplified by 28 cycles. Individual densities of both the target and the CC bands was obtained from each lane, and a ratio of NHE-2:CC was calculated. This ratio was therefore used to express the level of NHE-2 mRNA in this study. This data was further confirmed using a SYBR green real time PCR method.

### Real Time RT-PCR quantitation of NHE-2 mRNA

An aliquot of total RNA [1μg] was mixed with the primers PR1 and PR2 [100 pmoles of each], buffer and 1 unit of Platinum Taq DNA polymerase supplied with the kit [Promega]. Reactions were performed as follows: Reverse transcription: 42°C x 45 min → 30 [Denaturation: 94°C x 30 sec, Annealing: 45°C x 30 sec, Extension: 74°C x 60 sec]. A standard curve between the cycle threshold [CT] and concentrations of purified CC was prepared separately. The Corresponding CT values were obtained for both the non colitis control and the colitis RNA samples. The level of NHE-2 mRNA was calculated using the CT curve. A change in the mRNA was calculated with respect to the control CT values.

### Characterization of PCR fragment

The gel-purified PCR fragment was sequenced using a non-radioactive DNA sequencing method [Perkin-Elmer], with the downstream primer using a standard capillary gel electrophoresis method [3100 Avant Genetic Analyzer, ABI] in the RCF, Health Sciences Center, Kuwait University, Kuwait. Nucleotide sequence was identified using the Blast program (www.ncbi.nlm.nih.gov) with the published sequence.

### Heteronuclear RNA level

The mechanism underlying the regulation of NHE-2 mRNA expression was investigated by measuring the level of heteronuclear RNA [HnRNA] using RT-PCR. An aliquot [1μg] of total RNA was reverse transcribed using a single step RT-PCR kit. Specific primers PR4 and PR5 [Table 1] were used to amplify the level of HnRNA. These primers were selected from the adjacent exon and intron sequences corresponding respectively to 47172974–47172995 bp and 47173278–47173298 bp positions in the published gene sequence (NM_012653). The melting temperature for both primers was 58°C, and the expected size of PCR fragment was 325 bp [Table 1].

### Data analysis

Data is presented as mean±SEM. A nonparametric statistical analysis using analysis of variance and Student’s *t*-test for unpaired observations was used to calculate the significance, p. A value p<0.05 was considered statistically significant. The number [*n*] refers to the number of animals used in each experiment.

## Results

### Characterization of colitis

Colitis was characterized by measuring MPO activity, body weight, and colon hypertrophy, and microscopically by H&E and alcian blue staining.

### MPO activity

**Fig 1:**
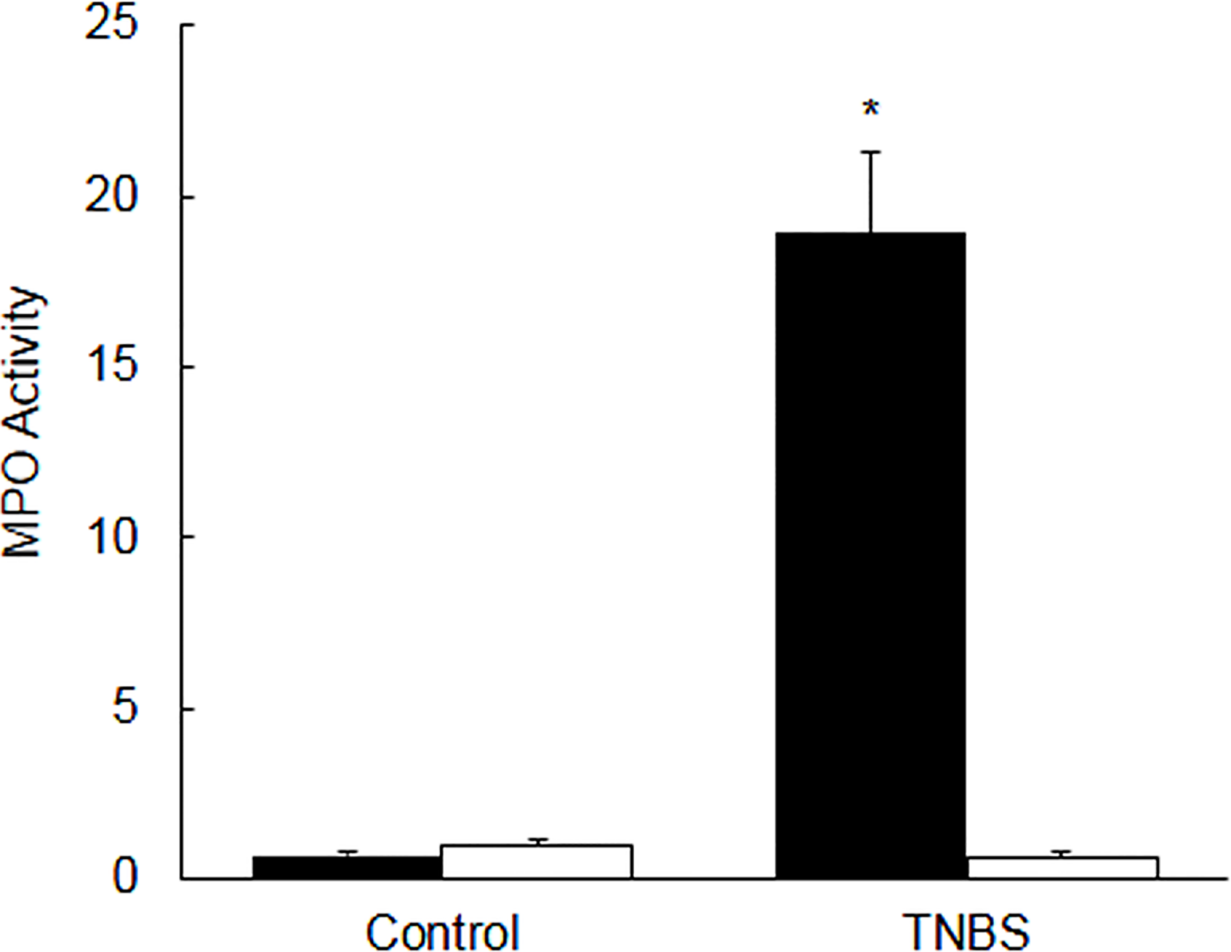
Bar diagram showing myeloperoxidase (MPO) activity as units per mg tissue in the colon (closed bars), and ileum (open bars) taken from the uninflamed (control) and inflamed rat colon on day 6 post-TNBS. Data are mean±SEM (n = 20). *Indicates significance p<0.05 between the control and TNBS.

In inflamed colon the level of MPO activity [units/mg tissue] was significantly [p<0.05] higher as compared to the non colitis control colon [Fig 1, closed bars]. On the contrary, the level of MPO activity in the ileum from the colitis rats was not different (p>0.05) as compared to that of the ileum from the non colitis controls [Fig 1, open bars].

### Body weight and colon hypertrophy

**Fig 2:**
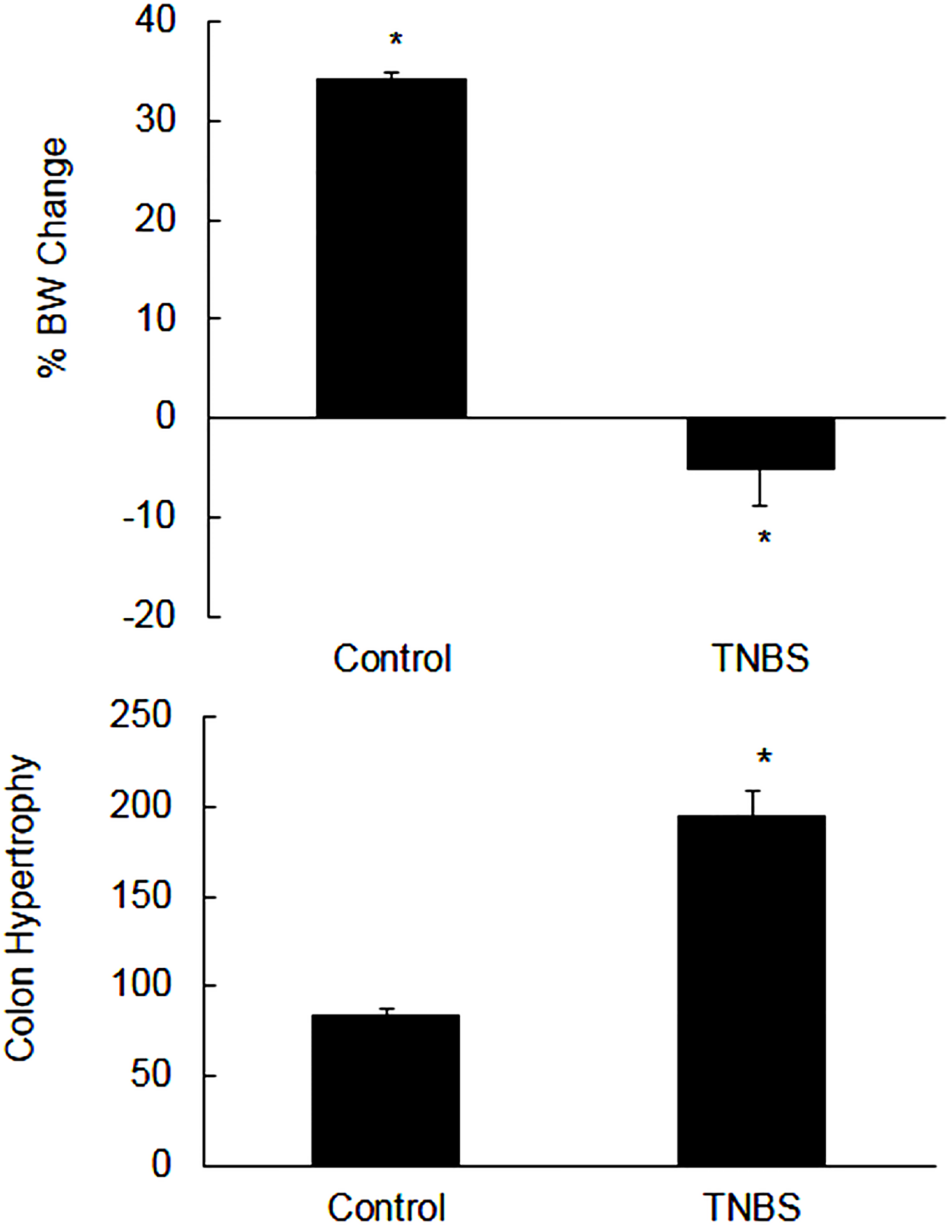
Upper panel: Shown are the percent changes in the body weight (BW) on day 6 post-TNBS as compared with their initial body weights at day 0. Data are mean±SEM (n = 20). *Indicates significance p<0.05 with respect to their initial weights at day 0. Lower panel: Bar diagram showing colon weight (mg) per cm length (hypertrophy) of the uninflamed (control) and inflamed rat colon on day 6 post-TNBS. Data are mean±SEM (n = 20). *Indicates significance p<0.05 versus non colitis control.

The colitis animals appeared to be sick, and lost weight significantly [p<0.05] as compared to their initial weights at day 0 [Fig 2, upper panel]. On the contrary, the non colitis control animals gained weight significantly [p<0.05] as compared to their initial weight at day 0 [Fig 2, upper panel].

The weights of colon in colitis were increased, and there was shortening of the colon length significantly [not shown]. Colon hypertrophy was expressed as weight [mg] per cm length of colon which was also significantly [p<0.05] higher in the inflamed colon as compared to the non colitis control colon [Fig 2, lower panel].

### Histochemistry and goblet cell staining

**Fig 3:**
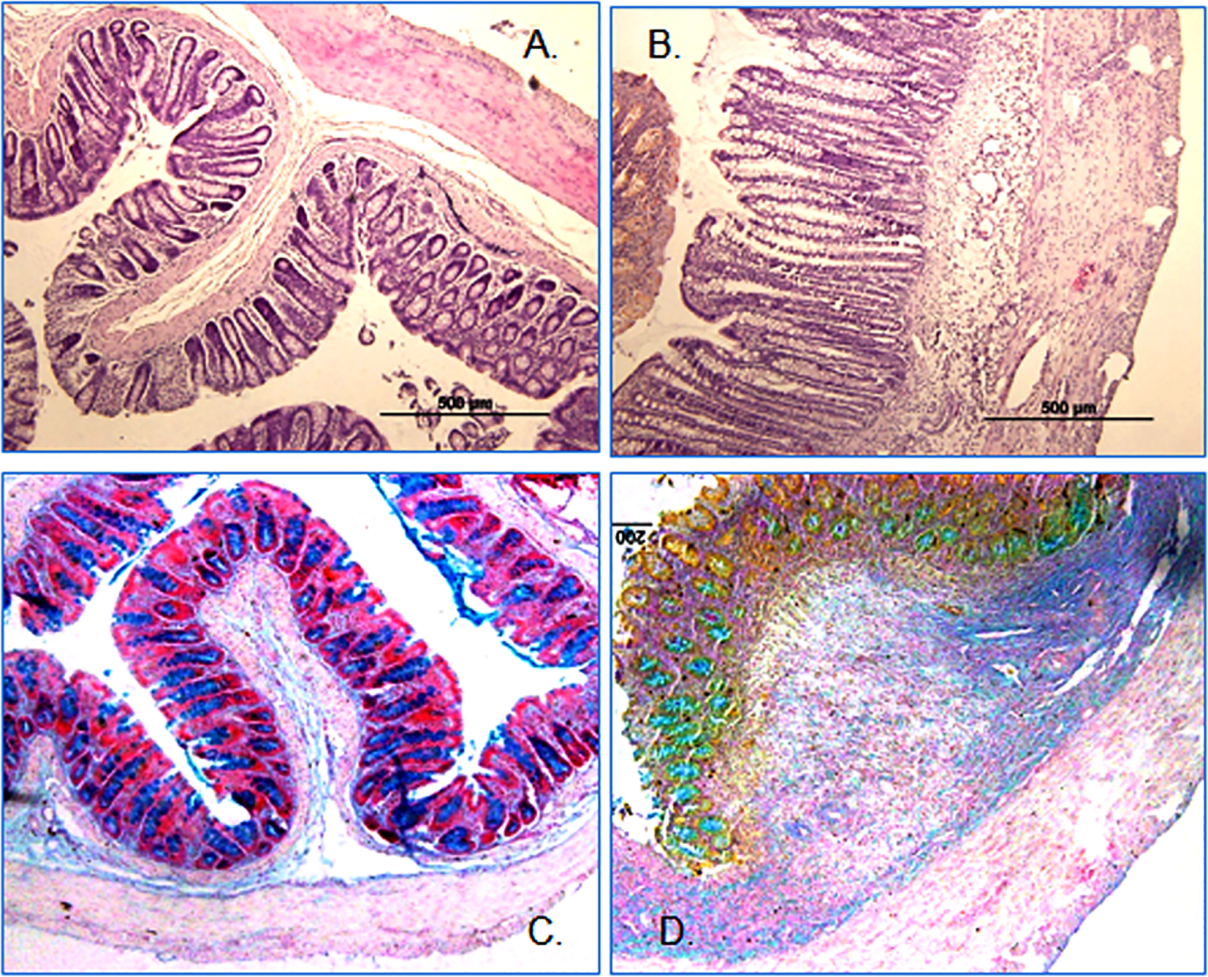
Representative picture (n = 9) showing H&E stained colon sections (A = Control, B = Colitis). Muscle hypertrophy and infiltration of inflammatory cells are evident. Goblet cells staining with Alcian blue dye of uninflamed colon [C] and inflamed colon [D]. A reduction of mucin expression is evident in the inflamed colon.

The H&E stained tissue sections revealed a significant infiltration of inflammatory cells and thickening of the muscle layers in inflamed colon. In contrary to the uninflamed control colon [Fig 3A], there were erosions in the epithelial cell lining and ulceration as well and an overall tissue histology deformation in the inflamed colon [Fig 3B]. Alcian blue staining was more prominent in the non colitis controls [Fig 3C] as compared to the inflamed colon [Fig 3D].

### Localization of NHE-2 and NHE-3 proteins

**Fig 4:**
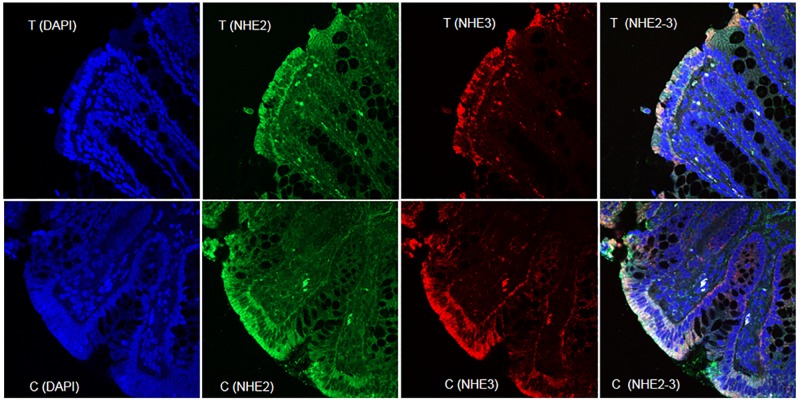
A representative confocal fluorescence microscopy picture showing expression of NHE-2 and NHE-3 and their colocalization in the epithelial brush borders of the control non inflamed and inflamed colon. Nuclei have been stained with DAPI. Magnification was 40x.

Both NHE-2 and NHE-3 proteins were colocalized on the brush borders in the epithelial cells lining the colonic lumen [Fig 4]. These NHE protein isoforms were not present in the cytoplasm, nucleus or on the basolateral domains [Fig 4]. A similar expression profile of both NHE-2 and NHE-3 isoforms was also evident in the inflamed colon [Fig 4]. The levels of both isoforms were also reduced in inflamed colon [Fig 4].

### NHE-2 protein expression

**Fig 5:**
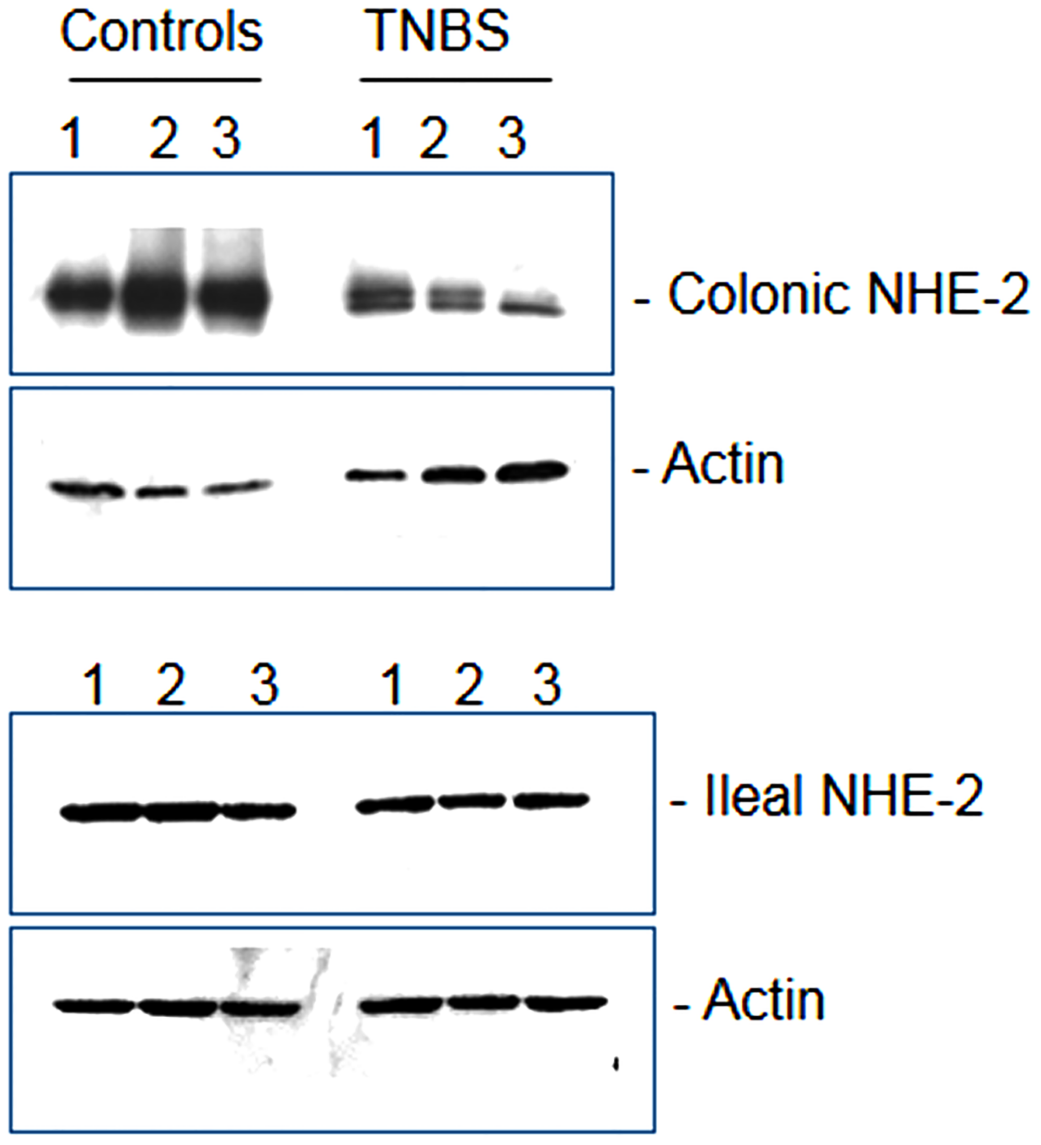
A representative ECL western blot picture showing expression of NHE-2 and actin proteins (n = 9) in three (1–3) controls and three (1–3) TNBS colitisin colonic (upper panel), and ileal (lower panel) segments.

**Fig 6:**
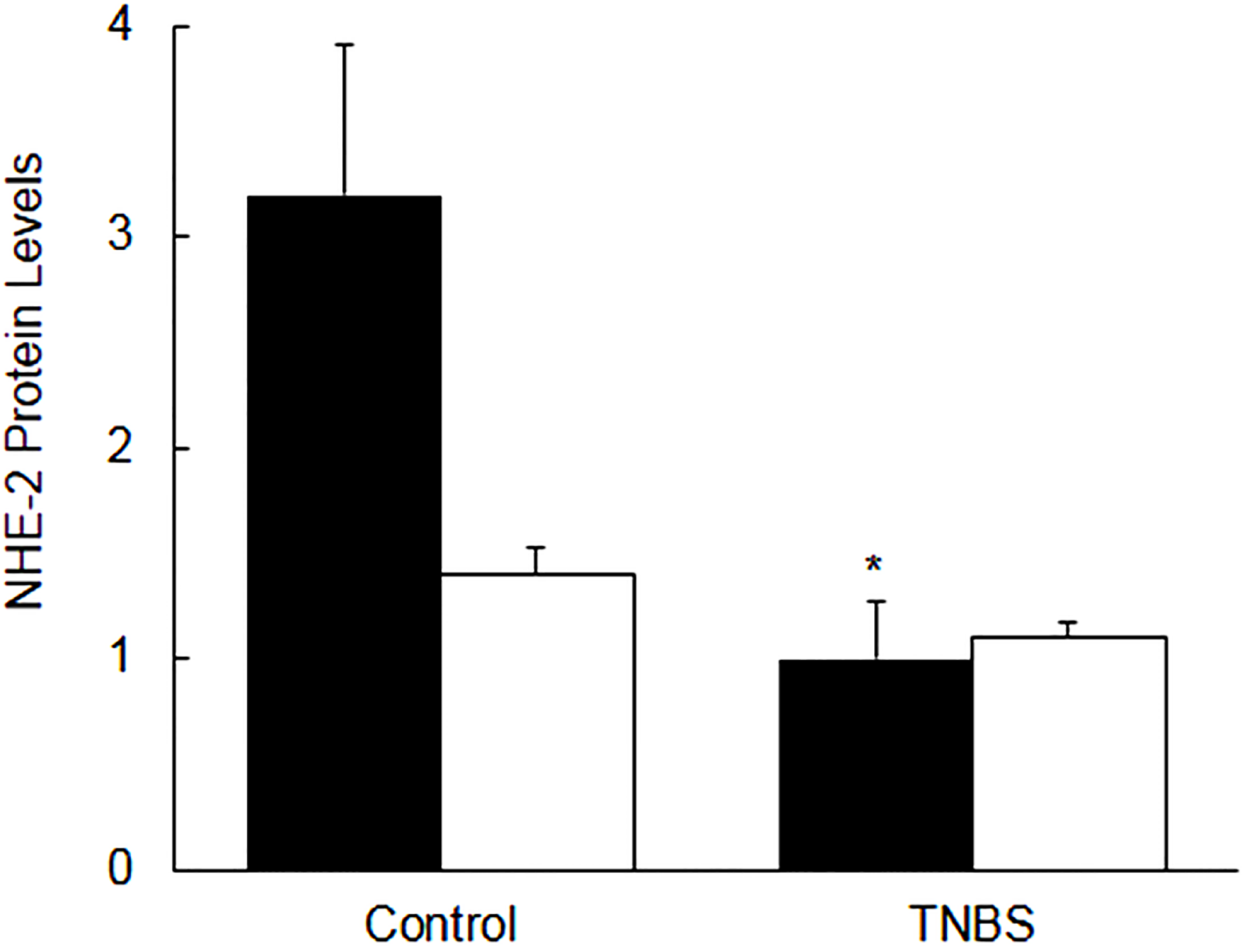
Bar diagrams showing the level of NHE-2 protein expression as a ratio NHE-2:actin in the control and TNBS–treated rat colon (closed bars) and Ileum (open bars). Data are mean±SEM (n = 9), *Indicates significance p<0.05) with respect to the controls.

The size of the reactive band confirmed separately corresponded to a molecular mass of 85–90 kD as expected [not shown]. ECL western blot analysis showed a significant reduction (p<0.05) in the steady state level of NHE-2 protein in the crude lysates from inflamed colon as compared to non-colitis controls [Figs 5 and 6, closed bars]. These changes were not reflected in the level of actin protein [Fig 5]. On the contrary, there was no significant alteration in the level of NHE-2 protein, or actin, in the ileum from colitis animals as compared with non colitic controls [Figs 5 and 6, open bars].

**Fig 7:**
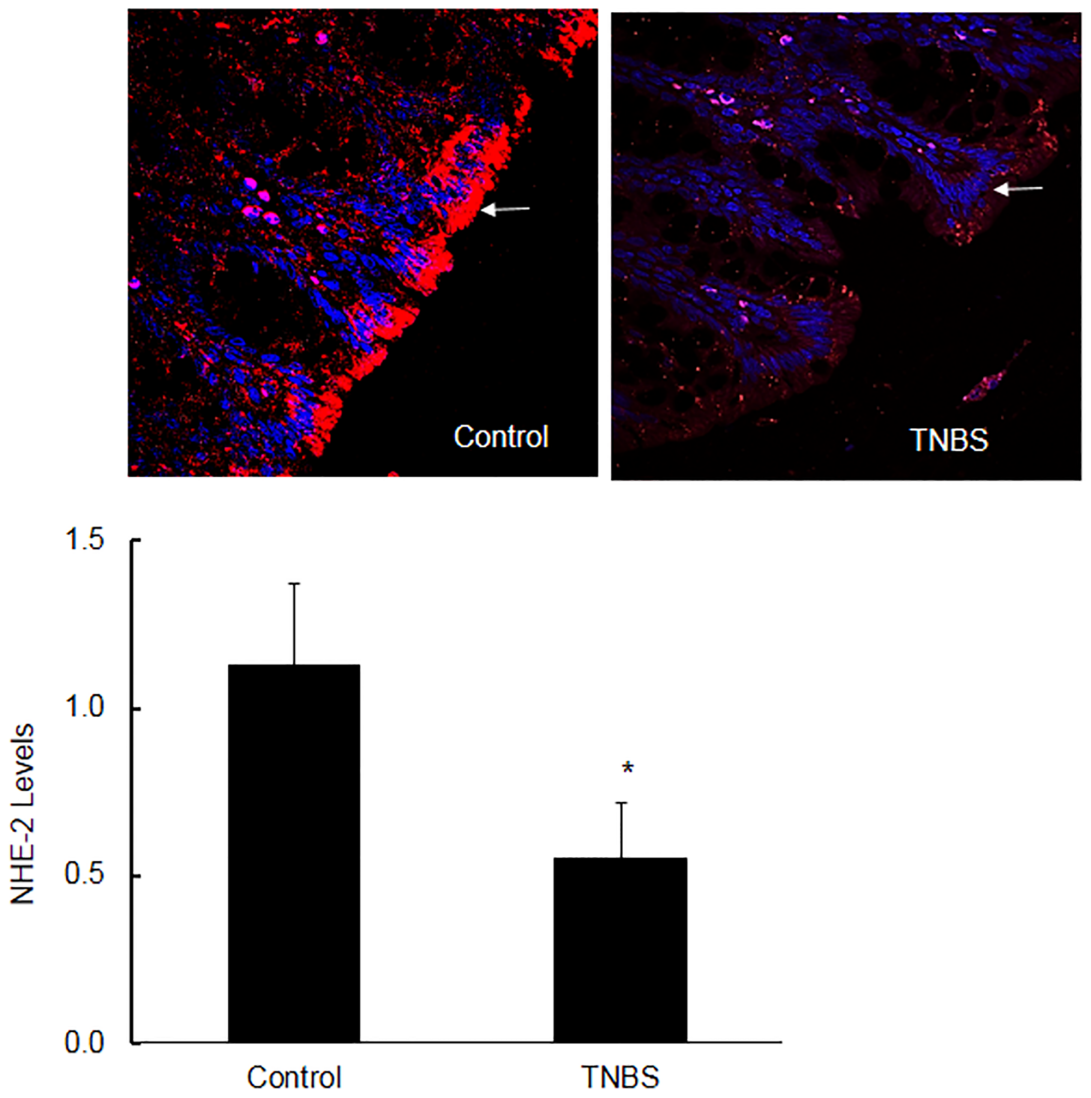
A representative (n = 9) confocal microscopy picture showing the levels of NHE-2 protein in the non colitic control and TNBS inflamed colon. Arrow indicates the location of NHE-2 expression (red) and blue dye indicates staining of the nuclei (DAPI). Magnification 40x (upper panel). Lower panel: Quantitative data shown as mean±SEM (n = 9). From each section 3–4 fields were selected for quantitation. *Indicates significance P<0.05 with respect to controls (lower panel).

Since the western blot analysis has multiple variables, it was necessary to support this data with confocal microscopy. The confocal data as shown in Fig 7, upper and lower panels were consistent with the ECL western blot analysis, indicating a significant [p<0.05] decrease in the NHE-2 protein levels in inflamed colon [Fig 7, upper and lower panels).

### NHE-2 mRNA expression

**Fig 8:**
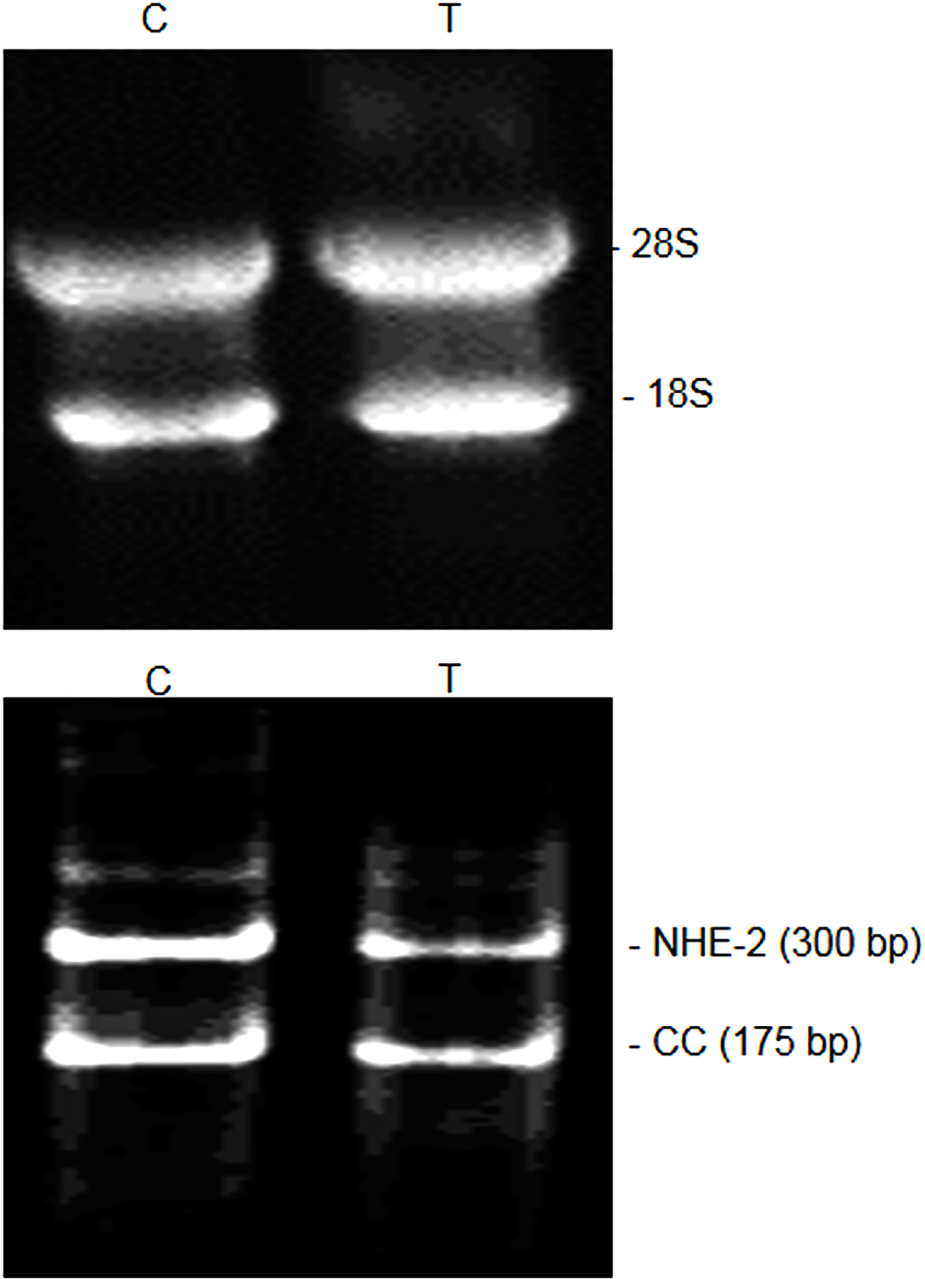
Agarose gel picture showing quality of total RNA extracted from the control (C) and colitis (T) colonic tissues used in this study. The 28S and 18S bands are ribosomal RNA (upper panel). Expression of NHE-2 mRNA (300 bp) and the competitive control (CC, 175 bp) in the control (C) and TNBS inflamed (T) colon using the end-point RT-PCR method (lower panel).

The quality and yield [μg/mg tissue] of total RNA were consistent in both the control (C) and colitis (T) samples [Table 2, Fig 8, upper panel]. The density of 28S and 18S on agarose gel and the LIN values indicated a similar quality of RNA in the control and TNBS-treated colon [Fig 8, upper panel, Table 2]. The NHE-2 mRNA [300 bp] and the CC [175 bp] PCR products were easily separated on the agarose gel or PAGE electrophoresis [Fig 8, lower panel]. Nucleotide sequence of the NHE-2 PCR fragment showed 100% identity with the published sequence [not shown]. In this study the level of mRNA expression was indicated as the ratios of NHE-2:CC.

**Table 2:** Protein and total RNA yields from the indicated tissues.

Conditions	Protein Yield (μg/mg tissue)		Total RNA Yield (μg/mg tissue)	RNA Quality LIN
	Colon	Ileum	Colon	Colon
Controls	28.4±1.1	63.5±4.9	2.52±0.5	7.0±0.5
Colitis	30.9±1.8	51.2±5.9	2.17±0.2	7.4±0.3
p	0.26	0.13	0.11	0.5

RNA quality in the control (uninflamed) and inflamed colon (colitis) measured in the arbitrary units (LIN) are shown as mean±SEM (n = 14). P values shown are non-significant with respective to the control in each case.

**Fig 9:**
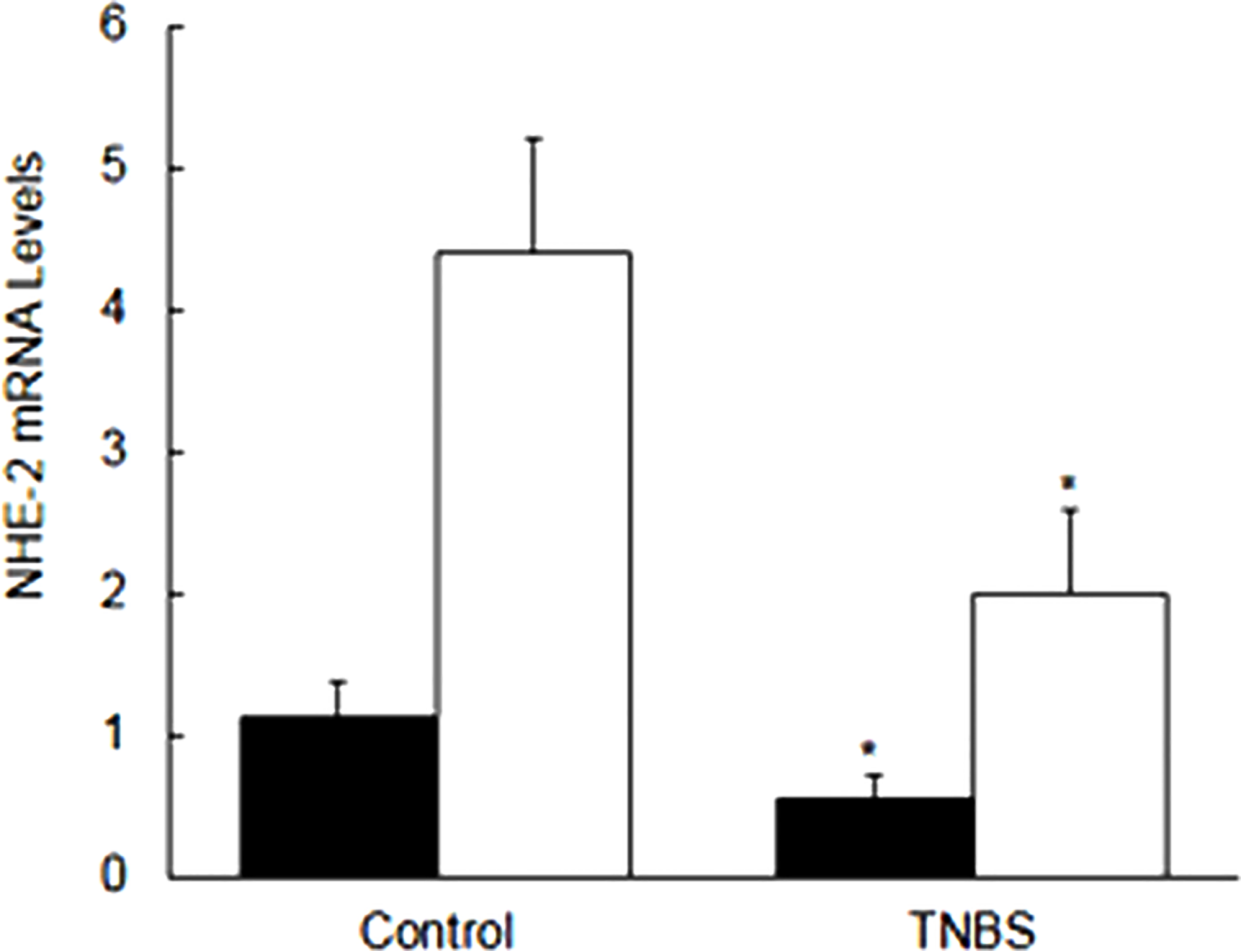
Bar diagram showing expression levels of mRNA as measured by the end-point RT-PCR (closed bars) and a SYBR green real time PCR (open bars). Quantitative expression level measured as ratios (NHE-2:CC) are shown. Data are mean±SEM (n = 9). *Indicates P<0.05 versus controls.

The level of NHE-2 mRNA measured using the end-point RT-PCR [Fig 9, open bars] was significantly [p<0.05] reduced in the inflamed colon as compared to the uninflamed control colon [Figs 8B and 9, open bars]. This data was further supported with the SYBR green real time PCR method [Fig 9, closed bars]. Similar to the end-point PCR amplification [Fig 9, open bars], results from the real time PCR [Fig 9, closed bars] also showed a comparable and a significant (p<0.05) reduction in the NHE-2 mRNA level [Fig 9] in inflamed colon [p<0.05].

### Expression of heteronuclear RNA

**Fig 10:**
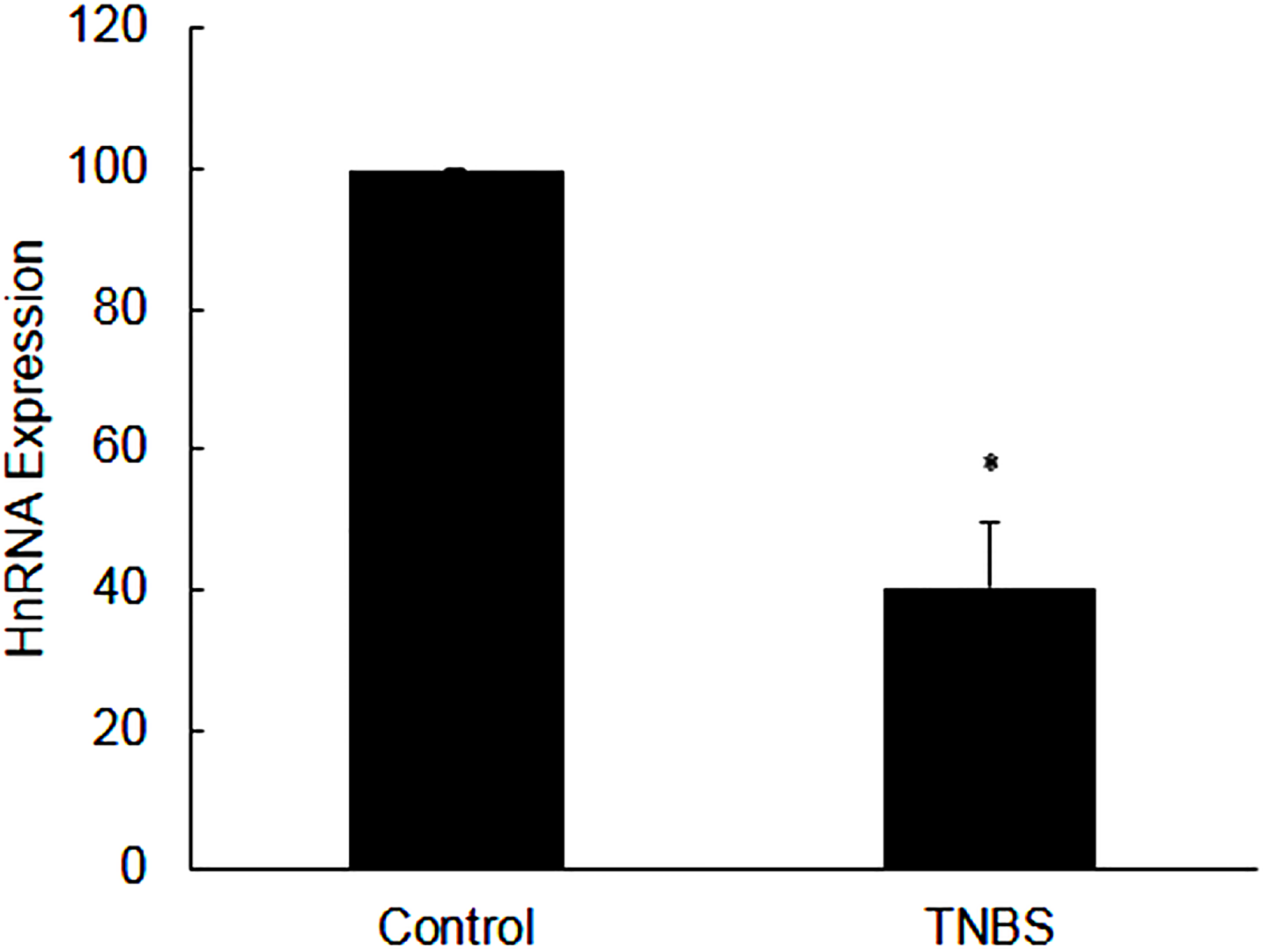
Bar diagram showing expression level of hnRNA as obtained from RT-PCR. Data are mean±SEM (n = 9). *Indicates P<0.05versus controls.

The level of HnRNA was also significantly decreased in inflamed colon [Fig 10]. This decrease in HnRNA level was not due to a genomic DNA contamination as there was no amplification in the negative control in which RNA was amplified without transcription reaction [Fig 10].

### *In vitro* Effect of TNF-α on the expression of NHE-2

TNF-α has been shown to suppress NHE-2 expression in the epithelial cell line through interaction with NFkB region present in the NHE-2 promoter region [17]. In our experimental condition, however treatment of colon with TNF-α *in vitro* did not show any difference in the expression of NHE-2 protein levels as compared to untreated controls [data not shown].

## Discussion

Diarrhea and altered GI-muscle contractility are common manifestations of inflammatory bowel diseases such as Crohn’s disease and ulcerative colitis. Cations such as Na^+^ and Ca^+2^ play an important role in maintaining these functions. Sodium is transported through multiple mechanisms including Na-H exchanger which is located in the plasma membrane and in the inner compartment of eukaryotic cells [1–4]. The isoforms NHE-2, -3 and -8 are present on the apical domain in the epithelial cells lining the GI-lumen. A strategic location of these isoforms suggests their role in the absorption of Na^+^ and water from dietary sources. Therefore a compromise in their expression should be expected to affect these processes and hence altered physiology. It is interesting to note that NHE-3 and -8 isoforms have been shown to be altered in both human IBD conditions and experimental colitis [22, 24–25]. From animal studies it is well known that NHE3 -/- knockout showed residual Na transport which is attributed to NHE-2 simply because the NHE-1 is highly resistant, and NHE-4 is highly sensitive to amiloride [16, 28]. Furthermore since NHE-2 is expressed abundantly in the intestinal brush border membrane and is regulated by immune stimuli [16, 28–30], it is expected to play a role in the pathogenesis of IBD. To address this issue we examined the expression of NHE-2 mRNA and protein in rat colon inflamed by TNBS, a well characterized animal model of colitis. The tissues used in this study are from animals which received TNBS 6 days earlier. This condition has been shown earlier to develop maximum inflammation in the current model. In the following paragraphs, the focus of the discussion will be on the characterization of colitis, expression of NHE-2 mRNA and protein and localization of NHE-2 in the rat colon, and finally an interpretation of data will be presented in relation to its role in the normal and pathophysiology of GI tract.

### Characterization of colitis

The colitis animals lost body weight significantly. The colon from TNBS treated animals was shortened and thickened significantly on the day 6 post-induction of colitis. Furthermore, there was a significant increase in the MPO activity in inflamed colon but not in the uninflamed ileum, suggesting that TNBS used in this study caused a localized inflammation in the colon. Thus this is considered as a model of Crohn’s disease and not of ulcerative colitis. There was an increased infiltration of inflammatory cells in the mucosa and muscle layers in the inflamed colon as well. Goblet cells’ staining with alcian blue dye demonstrated damage in the epithelium and a decrease in mucin production. Under normal conditions mucin makes a protective barrier which prevents sensitization of the GI tract by normal microflora or food antigens. These findings are consistent with previous reports, which taken together confirmed presence of inflammation in the tissues used in this study [22, 24–25].

### Expression and role of NHE-2

IBD is associated with loss of electrolyte and water that has been attributed in part to the suppression of NHE-3 and NHE-8 isoforms [22, 25]. However, the role of NHE-2 is not certain and was the focus of this study. To address this we examined the levels of NHE-2 protein, however before its quantitation we confirmed its co-localization with the NHE-3, an apical isoform which is known to express in the colonic epithelial cells. Localization data from confocal immunofluorescence demonstrated that NHE-2 is a prominent isoform located on the brush borders in the colon epithelial cells which suggests a prominent role in the NaCl absorption from the lumen. It is worth mentioning that the NHE-3 is known to shift its location to internal stores under certain conditions. However, the level of both the isoforms NHE-2 and -3 was decreased but retained the same localization on the brush borders (Fig 4), further support that this is due to decrease in the protein concentration, and not due to its translocation to internal stores. We and others have shown a decreased expression of NHE-3 in human IBD and in animal model of colitis and therefore in this study we did not present quantitative data specifically on the NHE-3 isoform [31–32]. With regard to NHE-2 protein estimation, since western blot analysis involves a large number of variables, therefore confocal immunofluorescence microscopy was also used to support our western blot results. Interestingly, confocal microscopy also showed a similar decrease in the NHE-2 protein levels in the inflamed colon. The decrease in the level of NHE-2 protein was selective as the level of actin, an internal control, remained unchanged. In addition, this decrease is also not a reflection of changes in the total protein contents as the protein yield remained similar in both the inflamed and uninflamed colon [Table 2]. Furthermore, these changes were not conspicuous in the non inflamed ileum of the colitis animals, which are suggestive of the fact that changes in colon are inflammation induced and localized to the inflamed colon only. Therefore further investigations on the non-inflamed ileum were not extended in this study. Physiologically these findings may be interpreted to compromise the absorption of NaCl and water hence contribute to the development of diarrhea in the present model of colitis. Furthermore, we have not measured the uptake of Na^+^ or water in this condition as this is a well-known fact that the electrolyte uptake is impaired in inflamed enterocytes which is a contributory factor to the development of diarrhea in these animals [20–21, 33– 34]. This has been reported that in DSS model, suppression of NHE-3 activity is accompanied by an induction of the NHE-2 activity as a counter act mechanism [34]. However, our findings do not support such changes as both the NHE-2 and NHE-3 isoforms were suppressed in the present model (Fig 4). In addition, expression of the apical isoforms NHE-3 and NHE-8 is also decreased in IBD [25, 32]. Thus our results are at variance with those reported earlier [34] which could be due to differences in the models used, degree and location of inflammation, and also the type of etiological agents used to induce inflammation.

### Mechanism of NHE-2 suppression

Next we investigated the mechanism of the decrease in NHE-2 protein expression which might involve pre- and post-transcriptional processes. Since the level of protein is usually directly correlated with the mRNA level, in this study we measured the level of NHE-2 mRNA using RT-PCR method. We used an end-point RT-PCR amplification (28 cycles) using a competitive control which was prepared in this study. These findings demonstrated a reduction in the level of NHE-2 mRNA in inflamed colon. To support these findings we further measured the mRNA levels by a SYBR green real time RT-PCR method. For this purpose, a standard curve between the cycle threshold [CT] and known concentrations of the competitive control was constructed to calculate the level of NHE-2 mRNA expression. Interestingly, the real time PCR findings also showed a similar reduction in the level of NHE-2 mRNA, suggesting that the decrease in NHE-2 protein levels is due to a decrease in the NHE-2 mRNA abundance in inflamed tissue. Furthermore, the yield and the integrity of total RNA used in this study were not different in the inflamed colon than the controls [Table 2]. Therefore this reduction in the NHE-2 mRNA level is not attributable to these factors. Thus the three apical isoforms NHE-2 (present study), NHE-3 [32] and NHE-8 [25] are suppressed in colitis which together contribute to the pathogenesis of IBD. However, the isoform selective inhibitors are currently not known, therefore quantitative contribution of the NHE-2 isoform remains to be investigated.

### Transcription of NHE-2

To investigate if transcription has a role in regulating the level of mRNA in inflamed tissue, we measured the level of heteronuclear RNA using the real time RT-PCR with SYBR green. For this purpose we used an upstream primer (PR4) selected from an intron, and a downstream primer (PR5) selected from an adjoining exon of NHE-2 gene sequence [Table 1]. We used the same competitive control in this case to measure HnRNA levels as well. Our findings demonstrate a significant reduction in the level of HnRNA in inflamed colon suggesting a role of transcription in the suppression of NHE-2 mRNA expression in inflamed colon. Analysis of the ratio of NHE-2 protein:NHE-2 mRNA levels demonstrated that this ratio was not statistically different in the inflamed colon as compared to the uninflamed control colon (data not shown). Therefore, these findings could suggest that the translation step is not compromised in this model, and support our contention that the transcription of the NHE-2 gene is a main regulatory process in inflamed colon in this model.

Studies using recombinant cells have shown that NHE-2 gene is immune responsive as it responds to TNF-α and INFγ *in vitro*. Since the level of these inflammatory mediators are induced in IBD [19, 20], we examined effects of TNF-α on the expression of NHE-2 using uninflamed colonic strips [14, 28–30]. The uninflamed colonic strips were treated with different concentrations of TNF-α, and the level of NHE-2 protein was measured. Our *ex vivo* findings discount a role of TNF-α in the suppression of NHE-2 in the experimental colitis (data not shown). Our findings are different than those reported earlier [17] which could be explained due to many factors. In our study we used the whole colonic segment while others have used cultured cell line or the animals injected with TNF-α. It is likely that other factors are required *in vivo* for the action of TNF-α as compared to the present *ex vivo* study. In addition, recombinant cells are different in many respects including that they carried a recombinant NHE-2 gene promoter [17] which was more likely to respond to TNF-α as against the *ex vivo* condition used in the present study. It is worth noting that TNF-α is commonly used to treat Crohn’s disease, and in this present model TNF-α and other cytokines have been shown to be upregulated [17–19].

In conclusion, taken together these findings suggest that NHE-2 is colocalized with NHE-3 on the apical domain of the colonic epithelial cells, and that NHE-2 is more abundant than the NHE-3 in the colonic epithelial cells. The decrease in NHE-2 expression together with NHE-3 and NHE-8 [25, 31–35] may contribute significantly to diarrhea through a compromised action on the absorption of NaCl from the colonic lumen. Additionally, the suppression of NHE-2 is regulated transcriptionally. However, due to unavailability of isoform selective inhibitors it remains to be known as to what extent the NHE-2 isoform contributes to electrolyte and water absorption and secretion in this model. These findings are new and add new insights into the pathogenesis of IBD with reference to a role of NHE-2, and hence may be taken into consideration while designing a new therapeutic approach for the treatment of IBD.
